# Supplementary material for: The Effectiveness and Safety of Long-Term Macrolide Therapy for COPD in Stable Status: A Systematic Review and Meta-Analysis
Source: Diseases. 2023 Oct 27;11(4):152. doi: 10.3390/diseases11040152 (PMC10660475; doi:10.3390/diseases11040152)

Supplementary Materials

Figure S1. Risk of bias graph of included studies

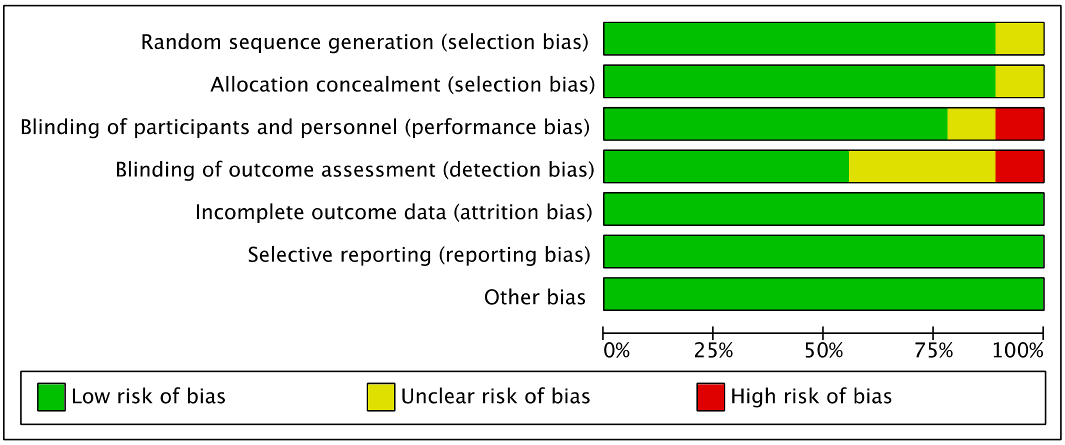

Figure S2. Suppression of exacerbations per patient-year

	Macrolide			Placebo				Mean Difference
Study or Subgroup	Mean	SD	Total	Mean	SD	Total	Weight	IV, Random, 95% CI
**2.2.1 Azithromycin**								
Albert 2011	1.48	0.32	558	1.83	0.25	559	55.8%	−0.35 [−0.38, −0.32]
Uzun 2014	1.94	1.78	47	3.22	2.31	45	10.7%	−1.28 [−2.13, −0.43]
**Subtotal (95% CI)**			**605**			**604**	**66.5%**	**−0.72 [−1.61, 0.17]**
Heterogeneity: Tau² = 0.34; Chi² = 4.64, df = 1 (P = 0.03); I² = 78%								
Test for overall effect: Z = 1.57 (P = 0.12)								
**2.2.2 Erythromycin**								
He 2010	0.61	0.39	16	1.11	0.55	15	33.5%	−0.50 [−0.84, −0.16]
**Subtotal (95% CI)**			**16**			**15**	**33.5%**	**−0.50 [−0.84, −0.16]**
Heterogeneity: Not applicable								
Test for overall effect: Z = 2.90 (P = 0.004)								
**Total (95% CI)**			**621**			**619**	**100.0%**	**−0.50 [−0.81, −0.19]**
Heterogeneity: Tau² = 0.04; Chi² = 5.38, df = 2 (P = 0.07); I² = 63%								
Test for overall effect: Z = 3.19 (P = 0.001)								
Test for subgroup differences: Chi² = 0.20, df = 1 (P = 0.66), I² = 0%								

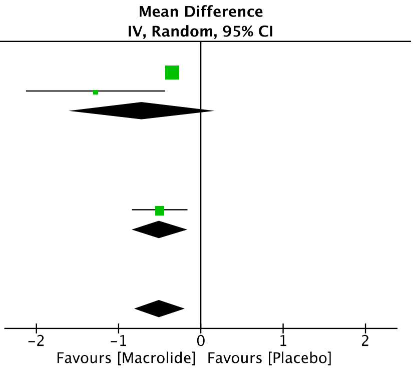

Figure S3. SGRQ of symptom, impact, and activity scores

SGRQ Symptom

Study or Subgroup	Experimental Mean	Experimental SD	Experimental Total	Control Mean	Control SD	Control Total	Weight	Mean Difference IV, Random, 95% CI
Albert 2011	-7	17.9	444	-3.7	16.5	453	86.7%	-3.30 [-5.55, -1.05]
Berkhof 2013	-9.2	18.2	37	0.1	18.2	37	6.4%	-9.30 [-17.59, -1.01]
He 2010	-2.5	14.8	18	0	8.92	18	6.9%	-2.50 [-10.48, 5.48]
**Total (95% CI)**			**499**			**508**	**100.0%**	**-3.63 [-5.73, -1.53]**

Heterogeneity: $Tau^2 = 0.00$; $Chi^2 = 1.95$, df = 2 (P = 0.38); $I^2 = 0\%$
Test for overall effect: Z = 3.39 (P = 0.0007)

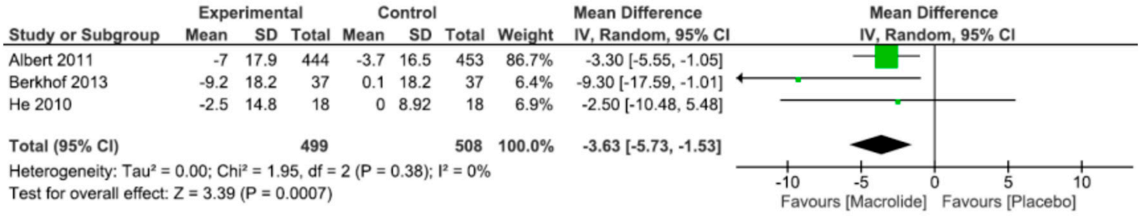

SGRQ Impact

Study or Subgroup	Experimental Mean	Experimental SD	Experimental Total	Control Mean	Control SD	Control Total	Weight	Mean Difference IV, Random, 95% CI
Albert 2011	-2.1	14.3	444	0	13.8	453	45.0%	-2.10 [-3.94, -0.26]
Berkhof 2013	-7.3	12.2	37	1.6	12.2	37	30.9%	-8.90 [-14.46, -3.34]
He 2010	2.8	15.4	18	0.2	4.7	18	24.1%	2.60 [-4.84, 10.04]
**Total (95% CI)**			**499**			**508**	**100.0%**	**-3.06 [-8.26, 2.14]**

Heterogeneity: $Tau^2 = 14.77$; $Chi^2 = 7.08$, df = 2 (P = 0.03); $I^2 = 72\%$
Test for overall effect: Z = 1.15 (P = 0.25)

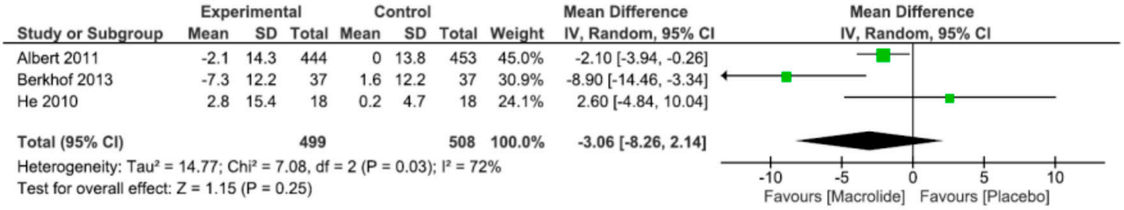

SGRQ Activity

Study or Subgroup	Experimental Mean	Experimental SD	Experimental Total	Control Mean	Control SD	Control Total	Weight	Mean Difference IV, Random, 95% CI
Albert 2011	-1.6	14.9	444	0.2	14.3	453	82.3%	-1.80 [-3.71, 0.11]
Berkhof 2013	-4.1	14	37	0.2	14	37	7.4%	-4.30 [-10.68, 2.08]
He 2010	-3.8	10.5	18	0.5	5.13	18	10.3%	-4.30 [-9.70, 1.10]
**Total (95% CI)**			**499**			**508**	**100.0%**	**-2.24 [-3.98, -0.51]**

Heterogeneity: $Tau^2 = 0.00$; $Chi^2 = 1.16$, df = 2 (P = 0.56); $I^2 = 0\%$
Test for overall effect: Z = 2.53 (P = 0.01)

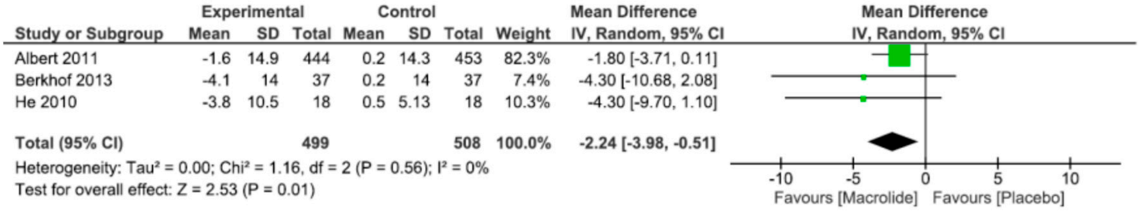

Figure S4. Adverse effects

Study or Subgroup	Experimental Events	Experimental Total	Control Events	Control Total	Weight	Odds Ratio M-H, Random, 95% CI
Albert 2011	182	558	155	559	95.0%	1.26 [0.98, 1.63]
Berkhof 2013	3	42	1	42	1.2%	3.15 [0.31, 31.62]
He 2010	2	16	3	15	1.6%	0.57 [0.08, 4.01]
Uzun 2014	5	47	2	45	2.2%	2.56 [0.47, 13.93]
**Total (95% CI)**		**663**		**661**	**100.0%**	**1.28 [1.00, 1.64]**
Total events	192		161			

Heterogeneity: $Tau^2 = 0.00$; $Chi^2 = 1.90$, df = 3 (P = 0.59); $I^2 = 0\%$
Test for overall effect: Z = 1.93 (P = 0.05)

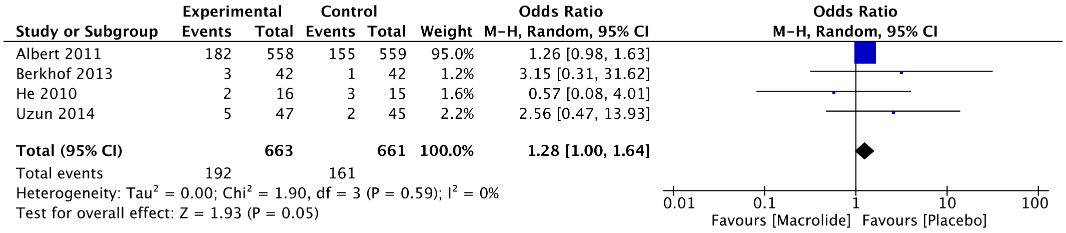

Supplement: Supplementary file 1 [file diseases-11-00152-s001.zip › diseases-2568654-supplementary.pdf]
